# The epidemiology of infant shaft fractures of femur or humerus by incidence, birth, accidents, and other causes

**DOI:** 10.1186/s12891-020-03856-4

**Published:** 2020-12-11

**Authors:** Johan von Heideken, Ingemar Thiblin, Ulf Högberg

**Affiliations:** 1 Department of Women’s and Children’s Health, Karolinska Institutet, 171 77 Stockholm, Sweden; 2 Forensic Medicine, Department of Surgical Sciences, Uppsala University, Uppsala, Sweden; 3 Department of Women’s and Children’s Health, Uppsala University, Uppsala, Sweden; 4 Epidemiology and Global Health, Umeå University, Umeå, Sweden

**Keywords:** Femoral shaft fracture, Humeral shaft fracture, Diaphyses, Infant, Birth injuries, Neonatal brachial plexus palsy, Accidents, Non-accidental trauma, Physical abuse

## Abstract

**Background:**

The purpose of this population-based registry study was to analyze both birth-related femur and humerus shaft fractures and diagnosed later in infancy, as regards incidence, perinatal characteristics, other diagnoses, and reported accidents.

**Methods:**

Children born in 1997–2014, diagnosed with a femur or humerus shaft fracture before age 1 year, were identified in the Swedish Health Registries. Rate of birth fractures were estimated by combining femur and humerus shaft fractures coded as birth-related with femur and humerus shaft fractures diagnosed during day 1–7 without registered trauma or abuse. Incidence was computed by comparing infants with femur or humerus shaft fractures to the total at-risk population.

**Results:**

The incidence for birth-related femur shaft fractures was 0.024 per 1000 children (*n* = 45) and that for birth-related humerus shaft fractures was 0.101 per 1000 children (*n* = 188). The incidence was 0.154 per 1000 children for later femur shaft fractures (*n* = 287) and 0.073 per 1000 children for later humerus shaft fractures (*n* = 142). Birth-related femur shaft fracture was associated with shoulder dystocia, cesarean, multiple birth, breech, preterm, and small-for-gestational age, while humerus shaft fracture was associated with maternal obesity, dystocic labor, shoulder dystocia, vacuum-assisted delivery, male sex, multiple birth, breech, preterm, large-for-gestational age, birth weight > 4000 g, and injury of brachial plexus. A bone fragility diagnosis was recorded in 5% of those with birth-related or later femur shaft fractures. Among infants with birth-related humerus shaft fractures, 1% had a bone fragility diagnosis; the figure for later fractures was 6%. Maltreatment diagnosis was associated with later fractures of both types, especially among those aged < 6 months, where approximately 20% (femur) and 14% (humerus) of cases, respectively, were associated with abuse. Fall accidents were reported in 73 and 56% among those with later femur and humerus shaft fractures, respectively.

**Conclusion:**

This study provides data on epidemiology, birth, parental characteristics, and reported accidents in relation to femur and humerus shaft fractures during infancy. Few children had a bone fragility diagnosis. Fall accidents were the main contributor to femur or humerus shaft fracture during infancy; however, the proportion of fractures attributed to maltreatment was high in children under 6 months.

**Supplementary Information:**

The online version contains supplementary material available at 10.1186/s12891-020-03856-4.

## Background

Shaft fractures of femur and humerus during infancy can occur in connection with birth or later. Birth-related shaft fractures of femur and humerus are rare and reported as case reports or small case series. A few studies, have reported birth-related femur shaft fracture rates from 0.13 per 1000 children (number of patients = 7) [1] to 0.14 per 1000 children (*n* = 4) [2] and birth-related humerus shaft fracture rates from 0.04 per 1000 children (*n* = 4) [3] to 0.06 per 1000 children (*n* = 2) [2]. Femur shaft fracture among infants, aged under 1 year, had an annual incidence of 0.16 per 1000 children (*n* = 313) in Sweden in the years 1987 to 2005 [4]. However, birth-related fractures were excluded from that population-based registry study. In a study analyzing treatment of femur shaft fractures in children aged 0 to 15 years of age (total study population *n* = 1852), Talbot et al. reported an incidence of 0.06 per 1000 children in children aged under 1 year [5]. The incidence of humerus shaft fractures during infancy has, to our knowledge, not been addressed before.

Adverse birth and infant characteristics reported for birth-related femur and humerus shaft fractures include: multiple birth, low birth weight, breech extraction, cesarean section, vacuum extraction, and fetal osteoporosis [1, 2, 6–8]. To the best of our knowledge, the relationships between maternal obesity or preeclampsia and shaft fractures of femur and humerus during infancy have not been studied. Shoulder dystocia has been associated with birth-related humerus fractures [9] and is a strong risk factor of brachial plexus birth palsy [10]. Preterm birth (< 37 weeks) has been associated with a 2.1–2.3-fold increased risk of future hospitalization for femur fractures before the age of 6 months [11, 12].

Most femur and humerus shaft fractures diagnosed later during infancy are related to fall accidents [4, 5, 13] but both bone fragility [12] and non-accidental trauma [4, 5, 14] are correlated in this age group. A recent large multicenter hospital study reported that one third of humerus fracture (excluding supracondylar fractures) and one fifth of femur fractures (to any part of the femur), in children under the age of 1 year, were associated with probable abuse [14].

We have previously, in a study not specifically addressing shaft fractures, reported the incidence of birth-related fractures for infants born in Sweden in 1997 to 2014, that diagnosis can be delayed, and that other fractures diagnosed among neonates might also be associated with birth [6]. The objective of this population-based registry study was to study both birth-related and later shaft fractures of femur or humerus during infancy, in children under the age of 1 year. The aim was to analyze the incidence of these fractures by sex, incidence, age at diagnosis, associations with prenatal exposure, birth complications, accidents, and other diagnoses during infancy.

## Methods

### Design

This was a nationwide population-based register study that comprised 1,855,267 infants born in Sweden between January 1997 and December 2014. We followed up the infants and their mothers until the children were 1 year of age using data from the National Patient Register (NPR), the Swedish Medical Birth Register (SMBR), and the Register of Children and Young Persons Subjected to Child Welfare Measures (RCWM) maintained by the Swedish National Board of Health and Welfare [15]. During the study period, the NPR used diagnosis codes from the Swedish version of International Classification of Diseases (ICD-10-SE).

### Cases

Among all children born during the 18-year period, we selected cases diagnosed with Birth injury to femur (P13.2), Birth injury to other long bones (P13.3), Fracture of shaft of femur (S72.3), and/or Fracture of shaft of humerus (S42.3) (Table S1).

#### Femur shaft fractures

**Fig. 1:**
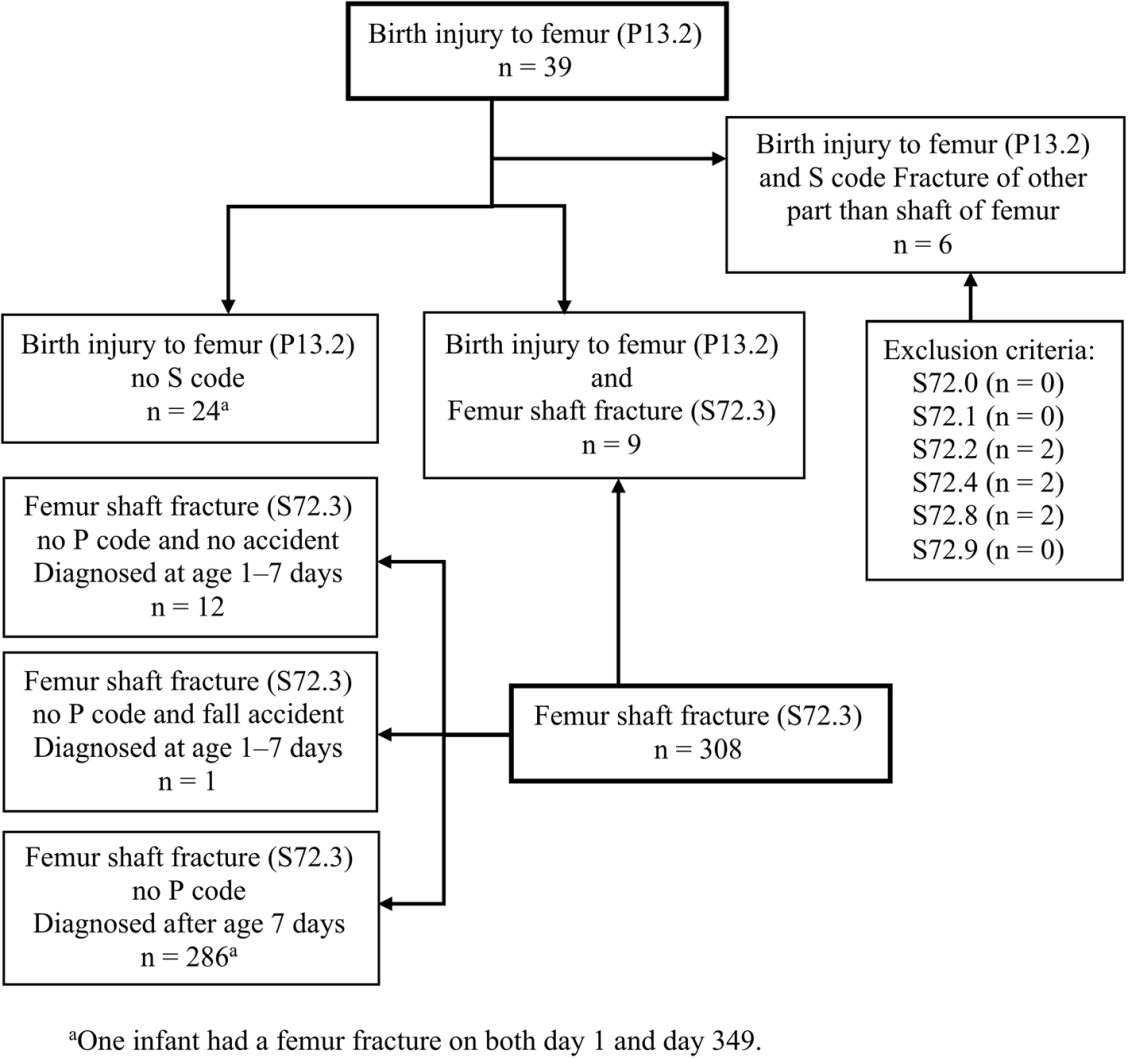
Study eligibility flowchart of children under the age of 1 year with femur fractures, identified in the National Patient Register

We found 39 infants with birth-related femur fractures (P13.2). Among them, 9 also had a femur shaft fracture diagnosis (S72.3), while 6 had S codes indicating fractures of other parts of the femur. We have assumed that the 24 birth-related femur fractures with no additional S code indicating which part of the femur was fractured were shaft fractures. One child had both a birth-related femur fracture and a femur shaft fracture on day of life 349 and was included in both of these categories. We found 299 infants with femur shaft fractures (S72.3) without a P code. Among them, 12 were diagnosed at the age of 1–7 days without a reported accident or abuse. We have assumed these fractures (27%) were also birth-related. These 45 cases formed the study base for birth-related femur shaft fractures. We found 287 infants diagnosed with femur shaft fractures (S72.3), without a presumed birth-related femur shaft fracture, and these cases formed the study base for femur shaft fractures diagnosed later in infancy (Fig. 1).

#### Humerus shaft fractures

**Fig. 2:**
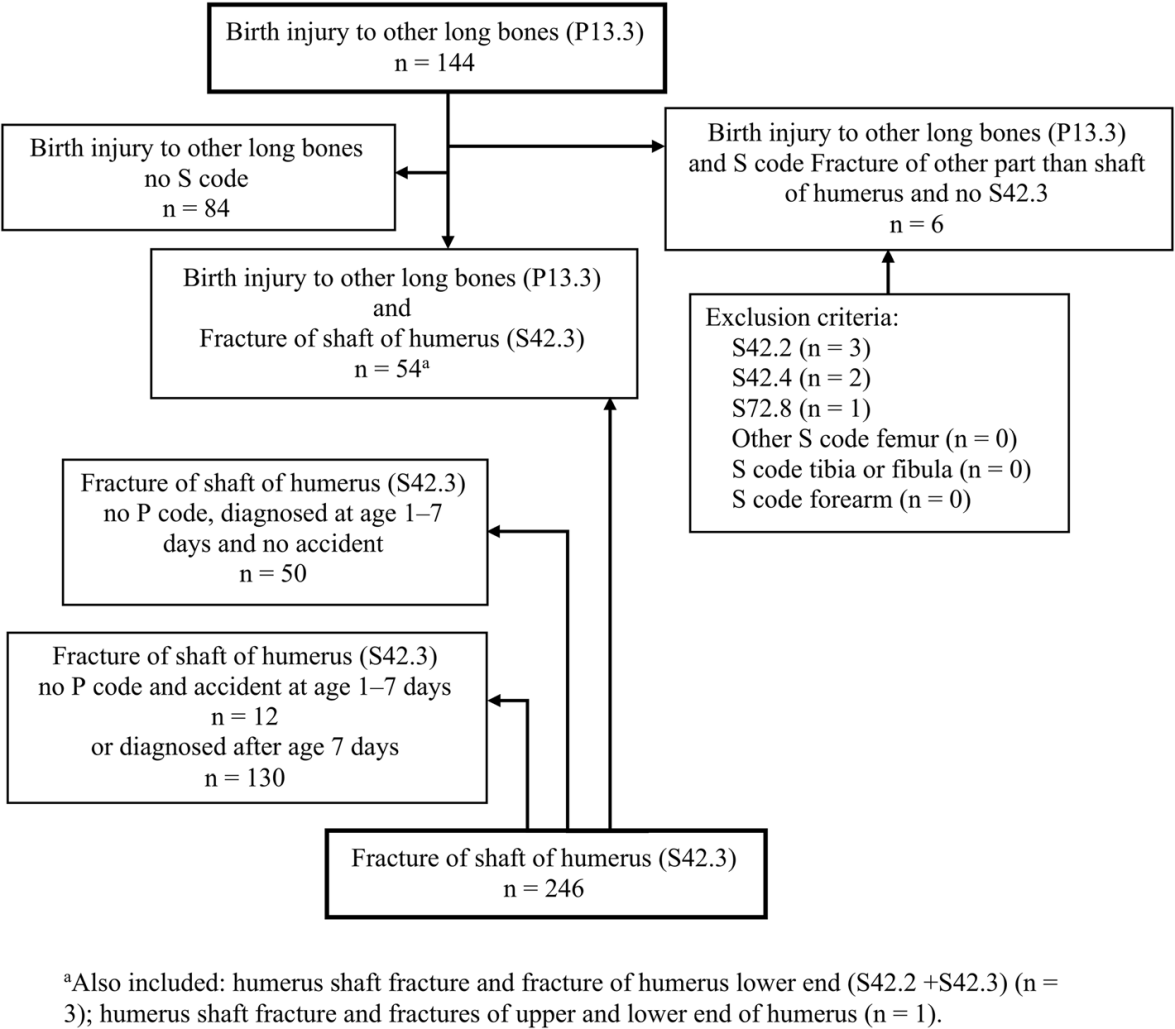
Study eligibility flowchart of children under the age of 1 year with humerus fractures, identified in the National Patient Register

We found 144 infants with birth-related long bone fractures (P13.3). Among them, 54 also had a humerus shaft fracture diagnosis (S42.3), 84 had no S codes indicating other long bone fractures, 4 had both a humerus shaft fracture and a fracture of proximal or distal humerus, and 6 had S codes indicating fractures of other parts than the humerus shaft. We found 192 infants with humerus shaft fractures (S42.3) without a P code; among them, 50 were diagnosed at the age of 1–7 days without a reported accident or abuse. We have presumed these fractures were birth-related and the estimated 188 cases formed the study base for birth-related humerus shaft fractures. We found 142 infants with humerus shaft fractures (S42.3), without a birth-related long bone fracture, and these cases formed the study base for humerus shaft fractures diagnosed later in infancy (Fig. 2).

### Population

Population controls were all infants without shaft fractures of humerus or femur; for maternal and perinatal exposure (SBMR), the control population was 908,324, and for other diagnoses (NPR) during infancy, the control population was 1,855,267, as described in a previous publication [12].

### Exposure

We categorized a number of exposures. The prenatal exposures included obesity (body mass index (BMI) at the start of pregnancy > 29.9 kg/m^2^) and preeclampsia. Delivery factors were dystocic labor and mode of delivery (spontaneous vaginal birth, vacuum-assisted, planned or emergency cesarean). The infant factors were sex, single or multiple birth, term or preterm birth (< 37 gestational weeks), birth weight (> 4000 g), small-for-gestational age (SGA) (< 2.5th or < 10th percentiles), and large-for-gestational age (LGA) (> 2.5th percentiles) accoding to Swedish definitions [15]. For SGA also the WHO-definition <10th percentiles was applied [16]. ICD codes for exposures, including accidental injury (± 7 days) and bone fragility, are defined in Table S1 (see Additional file 1). Bone fragility disorders were defined as having a diagnosis of osteogenesis imperfecta, rickets, or vitamin D deficiency before the age of 1 year. Maltreatment was defined as having a maltreatment diagnosis (Additional file 1) or an entry in the RCWM during the first year of life.

### Statistical analyses

Data are presented as descriptive statistics (mean and median). Time of birth-related fracture diagnosis during the first month was categorized into early or late neonatal period (1–7 days and 8–28 days). The age of each child with a later femur or humerus shaft fracture was categorized as < 6 months or 6–12 months. The incidence rate, incidence proportion, the proportion of cases per 1000 infants with 95% confidence intervals (CI), was computed by comparing the number of children with a fracture (ICD-10 code: S72.3, S42.3, P13.2, or P13.3) to the total number of children at risk. Fisher’s exact test was applied to assess differences. Statistical significance was defined as *P* < 0.05. To assess sex differences in relation to the categories of femur and humerus shaft fractures, crude and adjusted odds ratios were analyzed. For the statistical analyses, we used the statistical software package SPSS, version 25.0 (IBM Corp, New York, USA).

## Results

### Incidence, sex, and time of diagnosis

The incidence rate per 1000 live births for the estimated 45 infants with birth-related femur shaft fractures (60% boys, *p* value 0.535) was 0.024 [95% confidence interval (CI), 0.017–0.031] (Table 1). Fractures were registered during both the early (age 0–6 days) (*n* = 39) and late (age 7–27 days) (*n* = 4) neonatal period; however, 2 were registered after the age of 1 month. The incidence rate per 1000 children for the 287 infants with later femur shaft fractures (49% boys, *p* value 0.389) was 0.154 (95% CI, 0.138–0.170). Mean and median ages were 192 and 210 days. Over half (59%) were diagnosed during the sixth to twelfth month of life.

**Table 1:** Total number of femur and humerus shaft fractures, sex-specific, age-specific incidence rate per 1000 children and 95% confidence intervals (CIs). Crude odds ratio (OR) and adjusted OR for association between sex (girl reference) and femur and humerus shaft fractures by age group

Incidence per 1000 children (CI)				Crude OR (CI)	Adjusted^**a**^ OR (CI)
Age	Girls	Boys	Total		
*Femur shaft fracture*					
Birth-related	0.010 (0.005–0.014)	0.015 (0.009–0.020)	0.024 (0.017–0.031)	1.40 (0.77–2.55)	1.30 (0.66–2.58)
< 6 months	0.025 (0.018–0.032)	0.030 (0.022–0.037)	0.055 (0.045–0.065)	1.09 (0.74–1.62)	1.09 (0.72–1.64)
6–12 months	0.053 (0.043–0.064)	0.046 (0.037–0.056)	0.100 (0.086–0.113)	0.81 (0.61–1.08)	0.79 (0.59–1.07)
*Humerus shaft fracture*					
Birth-related	0.035 (0.027–0.043)	0.066 (0.055–0.078)	0.101 (0.087–0.115)	**1.81 (1.34–2.45)**	**1.68 (1.22–2.30)**
< 6 months	0.020 (0.014–0.027)	0.031 (0.023–0.039)	0.052 (0.042–0.062)	1.43 (0.95–2.15)	1.46 (0.96–2.23)
6–12 months	0.011 (0.006–0.016)	0.014 (0.082–0.019)	0.025 (0.018–0.032)	1.11 (0.62–1.99)	1.20 (0.65–2.22)

^a^Adjusted for large-for-gestational age (> 2.5th percentile), small-for-gestational age (< 2.5th percentile), preterm-born, shoulder dystocia

Significant odds ratios are written in bold

The incidence rate per 1000 children for the estimated 188 infants with birth-related humerus shaft fractures (66% boys, *p* value 0.000) was 0.101 (95% CI, 0.087–0.115); the adjusted odds ratio for the association between sex (girl reference) was 1.68 (95% CI, 1.22–2.30) (Table 1). Four of the cases had both a birth-related femur shaft fracture and humerus shaft fracture. None had a bone fragility disorder. Fractures were registered during both the early (*n* = 162) and late (*n* = 24) neonatal period; however, 2 were registered after the age of 1 month. The incidence rate per 1000 children for the 142 infants with later humerus shaft fractures (59% boys, *p* value 0.106) was 0.073 (95% CI, 0.065–0.089). Mean and median ages were 116 and 98 days. Over half (72%) were diagnosed during the first to fifth month of life.

### Maternal, birth and child characteristics

The infants with birth-related femur shaft fractures had several statistically significant differences in background characteristics compared with the general population, such as being born by shoulder dystocia (4%, *p* value 0.005), cesarean (74%, *p* value 0.000), multiple birth (20%, *p* value 0.000), born in breech (76%, *p* value 0.000), preterm (25%, *p* value 0.008), SGA < 10th percentiles (36%), SGA <  2.5th percentiles (12%, *p* value 0.008). However, no associations were noted for dystocic labor, vacuum-assisted delivery or infant sex. Among the later femur shaft fractures at age <  6 months, there was a significantly higher proportion of preeclampsia (7%, *p*-value 0.043), multiple birth (11%, *p* value 0.000), children born preterm (18%, *p* value 0.000), SGA < 10th percentiles (21%, *p* value 0.003), SGA <  2.5th percentiles (7%, *p* value 0.020) compared with the general population. Shaft fractures diagnosed at age 6 to 12 months had a higher proportion of maternal obesity (45%, *p* value 0.036) and SGA < 10th percentiles (16%, *p* value 0.018) (Table 2).

**Table 2:** Femur shaft fractures diagnosed among infants born in Sweden 1997 to 2014, birth-related (*n* = 45) or later (*n* = 287), by maternal, birth, and child characteristics, accidents, and other diagnoses

	Population (*n* = 908,324)	Femur shaft fracture					
		Birth-related (*n* = 45)		Later Age (months)			
				< 6 (*n* = 102)		6 to 12 (n = 185)	
	n (%)	n (%)	*p* value*	n (%)	*p* value*	n (%)	*p* value*
**Mother**							
Obesity	302,613 (37)	16^a^ (46)	.299	38 (43)	.324	75 (45)	.036
Preeclampsia	28,706 (3.2)	1 (2)	1.0	7 (7)	.043	10 (5)	.089
**Birth**							
Dystocic labor	107,225 (11.8)	4 (9)	.816	10 (10)	.646	18 (10)	.423
Shoulder dystocia	2005 (0.2)	2 (4)	.005	0		0	
Cesarean section	161,554 (18)	34 (74)	.000	30 (29)	.003	32 (17)	.915
Vacuum	62,099 (6.8)	4 (9)	.775	7 (7)	1.0	18 (10)	.267
Forceps	2473 (0.3)	0		1 (1)	.243	0	
**Child**							
Boy	469,344 (52)	27 (60)	.333	55 (54)	.723	86 (47)	.181
Multiple birth	26,639 (2.9)	9 (20)	.000	11 (11)	.000	10 (5)	.074
Breech	33,998 (3.3)	31 (76)	.000	10 (10)	.005	5 (3)	.564
Preterm	56,521 (6.2)	11 (25)	.008	18 (18)	.000	12 (7)	.878
SGA^b^ (< 10th percentile)	94,217 (10.4)	15 (36)	.000	21 (21)	.003	29 (16)	.018
SGA^b^ (< 2.5th percentile)	20,561 (2.3)	4 (12)	.008	6 (7)	.020	5 (3)	.610
LGA^c^ (> 2.5th percentile)	32,172 (3.7)	1 (3)	1.0	2 (2)	.776	6 (3)	1.0
Birth weight > 4000 g	169,517 (19)	5 (11)	.251	9 (9)	.013	33 (18)	.843
Injury of brachial plexus	1969 (0.1)	1 (2)	.093	0		0	
**Other diagnosis during infancy**							
Osteogenesis imperfecta	70 (0.004)^d^	2 (4)	.000	5 (5)	.000	8^e^ (4)	.000
Rickets or VDD^f^	259 (0.01)^d^	0		2 (2)	.000	1 (0.5)	.000
Maltreatment	1347 (0.07)^d^	0		21^g^ (20)	.000	6 (3)^h^	.000

^*^ Fisher’s test

^a^Missing (*n* = 10), ^b^Small-for-gestational age, ^c^Large-for-gestational age, ^d^Per 1,855,267, ^e^Entry in the Register of Children and Young Persons Subjected to Child Welfare Measures (*n* = 1) in connection with the fracture and OI diagnosis 137 days after the fracture, ^f^Vitamin D deficiency, ^g^Maltreatment diagnosis (*n* = 10), entry only in the Register of Children and Young Persons Subjected to Child Welfare Measures (*n* = 11); other diagnoses: subdural hemorrhage (*n* = 2), retinal hemorrhage (*n* = 1), rib fractures (*n* = 4), skull fractures (*n* = 2), ^h^Maltreatment diagnosis (*n* = 4), entry only in the Register of Children and Young Persons Subjected to Child Welfare Measures (*n* = 2)

Children with birth-related humerus shaft fractures had several statistically significant differences in background characteristics compared with the population. Half of the mothers had overweight/obesity. Adverse birth characteristics were maternal obesity (47%, *p* value 0.007), dystocic labor (23%, *p* value 0.000) shoulder dystocia (37%, *p* value 0.000), vacuum-assisted delivery (25%, *p* value 0.000), male sex (66%, *p* value 0.000), multiple birth (7%, *p* value 0.004), breech (10%, *p* value 0.000), preterm-born (13%, *p* value 0.001), birth weight of more than 4000 g (44%, *p* value 0.000), LGA (29%, *p* value 0.000) and injury of brachial plexus (24%, *p* value 0.000). Among the children with later humerus shaft fractures at age <  6 months, there was a significantly higher proportion of maternal obesity (54%, *p* value 0.001), shoulder dystocia (17%, *p* value 0.000), cesarean (26%, *p* value 0.040), LGA (14%, *p* value 0.001), birth weight of more than 4000 g (33%, *p* value 0.000), and injury of brachial plexus (18%, *p* value 0.000). Nine children with a diagnostic code for brachial plexus injury but without a reported accident were diagnosed with a humerus shaft fracture after 8 to 18 days. Few of those diagnosed at age 6 to 12 months had adverse perinatal characteristics (Table 3).

**Table 3:** Humerus shaft fractures among infants born in Sweden 1997 to 2014, birth-related (*n* = 188) and later (*n* = 142) by maternal, birth, and child characteristics, other fractures, and diagnoses

	Population (*n* = 908,324)	Humerus shaft fracture					
		Birth-related (*n* = 188)		Later Age in months			
				< 6 (*n* = 96)		6 to 12 (*n* = 46)	
	n (%)	n (%)	*p* value^*^	n (%)	*p* value^*^	n (%)	*p* value*
**Mother**							
Obesity	302,623 (37)	81^a^ (47)	.007	46 (54)	.001	21 (46)	.285
Preeclampsia	28,968 (3.2)	9 (5)	.205	4 (4)	.550	3 (7)	.177
**Birth**							
Dystocic labor	107,215 (11.8)	43 (23)	.000	12 (13)	.753	8 (17)	.249
Shoulder dystocia	2005 (0.2)	69 (37)	.000	16 (17)	.000	0	
Cesarean section	161,554 (18)	35 (19)	.782	25 (26)	.040	12 (26)	.174
Vacuum	69,101 (8)	47 (25)	.000	10 (10)	.331	2 (4)	.581
Forceps	2473 (0.3)	1 (0.5)	.401	1 (1)	.230	0	
**Child**							
Boy	469,339 (52)	123 (66)	.000	58 (60)	.106	25 (54)	.831
Multiple birth	26,645 (2.9)	13 (7)	.004	4 (4)	.369	5 (11)	.011
Breech	34,007 (3.3)	18 (10)	.000	5 (5)	.412	5 (11)	.028
Preterm	56,533 (6.2)	24 (13)	.001	9 (9)	.201	2 (4)	1.0
SGA^b^ (< 10th percentile)	94,244 (10.4)	16 (9)	.541	14 (15)	.181	9 (20)	.052
SGA^b^ (< 2.5th percentile)	20,566 (2.3)	3 (2)	.802	3 (3)	.478	4 (10)	.015
LGA^c^ (> 2.5th percentile)	32,141 (3.7)	50 (29)	.000	13 (14)	.001	0	
Birth weight > 4000 g	169,479 (19)	82 (44)	.000	32 (33)	.000	4 (9)	.089
Injury of brachial plexus	1969 (0.1)	45 (24)	.000	17^d^ (18)	.000	0	
**Other diagnosis during infancy**							
Osteogenesis imperfecta	76 (0.004)^e^	1 (0.5)	.017	3 (3)	.000	4^f^ (9)	.000
Rickets or VDD^g^	261 (0.01)^e^	1 (0.5)	.052	1 (1)	.000	0	
Maltreatment	1347 (0.07)^e^	0		12^h^ (14)	.000	2^i^ (4)	.000

^*^ Fisher’s test

^a^17 missing BMI, ^b^Small-for-gestational age, ^c^Large-for-gestational age, ^d^ No reported accident (*n* = 8), ^e^Per 1,855,267, ^f^Entry in the Register of Children and Young Persons Subjected to Child Welfare Measures (n = 1) in connection with the fracture and OI diagnosis 21 days after the fracture, ^g^Vitamin D deficiency, ^h^Maltreatment diagnosis (*n* = 9), entry in the Register of Children and Young Persons Subjected to Child Welfare Measures (*n* = 3); other diagnoses: subdural hemorrhage (*n* = 1), retinal hemorrhage (*n* = 1), rib fractures (*n* = 2), skull fractures (*n* = 1), ^i^Maltreatment diagnosis (*n* = 2), entry only in the Register of Children and Young Persons Subjected to Child Welfare Measures (*n* = 0)

### Osteogenesis imperfecta, rickets, vitamin D deficiency, abuse, and accidents

Two (4%) of the infants with a birth-related femur shaft fracture had osteogenesis imperfecta (OI). Neither had a later maltreatment diagnosis. Among later femur shaft fractures, 13 (5%) had OI; three of those cases were diagnosed 61 to 137 days after the fracture, of which one had a maltreatment diagnosis before the OI diagnosis. Also among later femur shaft fractures, 3 (1%) had rickets/vitamin D deficiency, one of which was diagnosed 97 days after the fracture; none of these children had a maltreatment diagnosis. Among the infants with later femur shaft fractures, 27 (9%) had a maltreatment diagnosis or entry in the RCWM. Among children < 6 months, 20% (*n* = 21) of cases were associated with abuse; other diagnoses among those were subdural hemorrhage (*n* = 2), retinal hemorrhage (*n* = 1), rib fractures (*n* = 4), and skull fractures (*n* = 2). A fall was registered as the cause of injury in 69% of the later femur shaft fractures; among specified fall accidents, a fall from being carried was the most common external cause (Table 4).

**Table 4:** Reported accidents causing later shaft fractures of femur or humerus

	No reported accident	Reported accident									
		Fall accident								Other type of accident	
		All	All	From being carried	From same level	From furniture or equipment	From bed	From stairs	Unspecified	Pinching	Transport
			n (%)	n (%)	n (%)	n (%)	n (%)	n (%)	n (%)	n (%)	n (%)
*Femur shaft fracture*											
< 6 months (*n* = 102)	37 (36.3)	65 (63.7)	53 (51.9)	16 (15.7)	5 (4.9)	4 (3.9)	3 (2.9)	0	25 (24.5)	10 (9.8)	2 (2.0)
6 to 12 months (*n* = 185)	30 (16.2)	155 (83.8)	145 (78.4)	34 (18.4)	24 (13.0)	20 (10.8)	13 (7.0)	14 (7.6)	40 (21.6)	8 (4.3)	2 (1.1)
All (*n* = 287)	67 (23.3)	220 (76.7)	198 (69.0)	50 (17.4)	29 (10.1)	24 (8.4)	16 (5.6)	14 (4.9)	65 (22.6)	18 (6.3)	4 (1.4)
*Humerus shaft fracture*											
< 6 months (*n* = 96)	48 (50.0)	48 (49.9)	44 (45.8)	8 (8.3)	1 (1.0)	3 (3.1)	2 (2.1)	0	30 (31.3)	3 (3.1)	1 (1.0)
6 to 12 months (*n* = 46)	11 (23.9)	35 (76.1)	35 (761.1)	3 (6.5)	9 (19.6)	7 (15.2)	2 (4.4)	0	14 (30.4)	0	0
All (*n* = 142)	59 (41.6)	83 (58.4)	79 (55.5)	11 (7.7)	10 (7.0)	10 (7.0)	4 (2.8)	0	44 (31.0)	3 (2.1)	1 (0.7)

Out of the 188 infants with birth-related humerus shaft fractures, 1 had OI and 1 had rickets/vitamin D deficiency. None of these children had a later maltreatment diagnosis. Among children with later humerus shaft fractures, 7 (5%) had OI – one of whom had a maltreatment diagnosis before the OI diagnosis – and 1 (1%) had rickets/ vitamin D deficiency. Out of the later humerus shaft fractures, 14 (10%) had a maltreatment diagnosis or entry in the RCWM; 12 (14%) were diagnosed before the age of 6 months and other diagnoses among those were subdural hemorrhage (*n* = 1), retinal hemorrhage (*n* = 1), rib fractures (*n* = 2), and skull fracture (*n* = 1). A fall was registered as the cause of injury in 56% of later humerus shaft fractures; a fall from same level or from furniture was the most common specified external cause (Table 4). Five of the later cases had concomitant femur shaft fracture and humerus shaft fracture. Three of had fall accidents, 2 had osteogenesis imperfecta. None had a diagnosis of abuse.

## Discussion

To our knowledge, this is the first study addressing the epidemiology of shaft fractures of femur and humerus, studying both birth-related and later fractures during the first year of life, with account taken of association to perinatal characteristics. As far as we know, this is also the largest population-based report on such fractures, and the first report on the incidence of humerus shaft fractures diagnosed during infancy but after birth.

The overall incidence of birth-related shaft fractures of femur and humerus in the present study was 0.024 and 0.101 per 1000 children, respectively. These results differ substantially from previously reported hospital case studies with incidence rates of 0.13–0.14 per 1000 children [1, 2] and 0.04–0.06 per 1000 children [2, 3]. Shaw et al. [13] reported that 20% (*n* = 5) of humerus shaft fractures in children under the age of 1 year were birth-related, compared with 60% in our study. It is unlikely that previous studies have had hidden cases, while hidden cases may exist with our register design. However, both the small sample sizes of previous reports [1–3, 13] and the differing settings might have contributed to the discrepancies compared with our population-based findings. Another reason could be our definition of birth-related fractures, which also included fractures identified in the early neonatal period without reported accidents or abuse, but not coded as birth-related. The existence of such cases indicates a possible underestimation of shaft fractures that might be associated with birth.

One of the issues emerging from this study is the challenges related to epidemiologic surveillance of birth-related femur and humerus shaft fractures, especially birth-related long bone fractures. The current P codes of birth injury to the skeleton in ICD-10 encompass the categories femur fractures and other long bone fractures. The ICD-11 released in 2018 has only one category: Birth injury to long bones (KA45.6). Our findings support the use of double coding, i.e., adding S codes, for improved precision of shaft diagnoses. Diagnosies of birth-related fractures might be delayed for femur fractures, [1, 17] especially among the preterm-born, [18] and for humerus shaft fractures [3, 8, 13]. We have previously reported that some birth-related fractures of femur or other long bones are diagnosed after the first week of life and in some cases at even higher age [6]. Our findings show an overlap between birth-related femur fractures and other long bone fractures. Whether these double diagnoses are due to coding errors, the identification of a cause, or delayed diagnosis cannot be ascertained, due to the study design with no access to patient records. In a clinical perspective, assessment of whether a fracture is birth-related or not has less importance for treatment; however, if the diagnosis is delayed and abuse is a potential cause, this can be of greater importance [8, 19].

The incidence of femur shaft fractures not related to birth found in this study (0.154 per 1000 children), more during last 6 months of the first year, was in line with previous findings in Sweden [4]. This is higher than what was reported from the UK by Talbot et al.; however, the authors stated that there might be an underestimation of the incidence due to missed cases in their dataset obtained from the Trauma and Audit Research Network and exclusion of children with open fractures and associated injuries [5]. In these previous studies, the risk of a femur shaft fracture was reported as higher among boys than among girls after the age of 1 year, but neither this study nor these past studies found any statistically significant sex differences during infancy [4, 5]. We cannot explain why boys in our cohort had a higher risk of birth-related diaphyseal humerus fractures but not of femur shaft fractures. Sex is not always reported but – interestingly – birth-related humerus shaft fractures previously reported by Basha et al. [2] and Sherr-Lurie et al. [3] were all in boys.

The current study found an incidence of later diagnosed humerus shaft fractures of 0.073 per 1000 children, being more prevalent during first 6 months of first year. A previous hospital study reported that among 101 children younger than 12 months treated at an emergency clinic, 2 patients were diagnosed with a humerus shaft fracture [20]. Another study reported an incidence of 0.17 per 1000 children (*n* = 3) in children aged < 24 months [21].

Our cases shared perinatal risk factors confirming the state of knowledge. For femur shaft fracture, these factors were breech presentation [1, 2, 6], multiple birth, and prematurity [1, 6], and for humerus shaft fractures, they were vacuum-assisted delivery, shoulder dystocia, and heavy birth weight, as reported for other birth-related long bone fractures [6]. A high proportion of cesarean delivery in connection with humerus shaft fractures [1–3] was not evident from our findings. Birth-related humerus shaft fractures and injuries of brachial plexus are known to occur in the setting of shoulder dystocia [9, 10]. Al-Rajeh et al. [22] reported that 11% (6 out of 57) children with congenital brachial palsy had an associated humerus fracture while Evan-Jones et al. [23] reported 2% (8 of 323). To our knowledge, this is the first study to document the coexistense rate of plexus injuries in infants with femur and humerus shaft fractures. However, there is a possibility that the true porportion in the present study is higher to a possible missclasification of the nine children with a diagnostic code for brachial plexus injury but with out a reported accident was classified based on day of diagnosis as later humerus shaft fracture.

Among those with a femur shaft fracture during the first to fifth month, we found a preponderance of the perinatal risk factors breech, prematurity, and SGA, and among those with humerus shaft fracture, shoulder dystocia, and heavy birth weight. Further, we found a significant association of preeclampsia to femur shaft fracture during first to fifth month, probably mediated through prematurity and SGA. Few perinatal risk factors were seen among those with femur or humerus shaft fractures diagnosed from the sixth month of life. For the early femur shaft fractures, this might be interpreted as a sign of bone fragility; however, this cannot be ascertained, due to the study design. We have previously shown that long bone fractures, not specifically shaft fractures, among infants of less than 6 months of age, especially those without accidents reported, are associated with risk factors for a metabolic bone disease, such as being born preterm [12]. Case reports have described rickets as being associated with humerus shaft fracture [24, 25] and femur shaft fractures [25]. Case series have reported long bone shaft fractures associated with metabolic bone disease of infancy [26]. We found only few shaft fractures with a diagnosis of rickets or vitamin D deficiency, as previously reported for fractures of any type [27].

Femur and humerus shaft fractures are closely associated with abuse diagnosis among infants underlining the importance of this differential diagnostic consideration. Our proportion of femur shaft fractures attributed to abuse, 9% (27 out of 287) with a preponderance at age below 6 months of 20% (21 out of 101), is higher than in the Swedish nationwide study, which reported a figure of 4.2% [4] probably because we included those having an entry in the RCWM. These numbers can be compared with those in previous studies reporting that 21–31% of femur fractures (including fractures of the entire femur) in children under the age of 1 year were caused by child abuse [14, 28]. Shaft fractures of the humerus are also common in connection with abuse [29]. Our outcome of 10% (14 out of 132), with a preponderance at age below 6 months of 14% (12 out of 96), of shaft humerus fractures having been assessed as abuse is lower than in a U.S. hospital study and a UK multicenter hospital study, which both reported that one third of humerus shaft fracture in children under the age of 1 year were associated with probable or indeterminate abuse [13, 14]. Since the prescence or non-prescence of other abuse suspected findings, and the diagnostic procedures regarding bone fragility cannot be dermined from the available registry data, it is unclear whether this discrepancy is due to different criteria for determination of abuse or a true difference because of different settings.

Eight out of 10 children with a femur shaft fracture occurring later in infancy had a reported accident; the vast majority had a reported fall accident as described earlier [4, 5], usually occurring in children aged 6–12 months. Our finding of 58% of children with a humerus shaft fracture having an accident reported is higher than the 29% reported by a hospital study (7 out of 24) [13]. However, much like in the study on femur shaft fractures by Talbot et al. [5], 29% of the children in our study had no reported accident.

In case of no reported accidents or in the absence of other abuse-suspected findings, e.g., bruises, warrants a thorough risk factor analysis, including perinatal exposure and bone fragility disorders. This is underlined by the fact that routinely used radiological methods have low sensitivity for detecting low bone mineralization [25]. Tests for metabolic bone disease such as measuring levels of calcium, phosphorus, alkaline phosphatase (ALP), parathyroid hormone (PTH), vitamin D, copper, and ceruloplasmin should be done routinely [26], and testing for genetic disease, e.g., osteogenesis imperfecta, should be considered.

### Strengths and limitations

A major strength of this study was the population design derived from diagnoses in a national patient registry considered to have high validity [30]. Although shaft fractures in infants have not been specifically validated in the patient registry, this fracture type can be considered to have high precision, although there is still the potential for misclassification bias or missing data. A strength was also the linkage to the RCWM to detect cases associated with abuse; we found cases that had no diagnosis of maltreatment, but had an entry in the out-of-home care register. A further strength was the linkage to a birth registry [31]. The rate of birth fractures was estimated by combining femur and humerus shaft fractures diagnosed in days 1–7 and fractures diagnosed as birth-related, in the absence of accidents and abuse in the first week of life. Our estimate of birth-related fractures might be underestimated, especially regarding humerus shaft fractures considering that birth weight of more than 4000 g, shoulder dystocia, and brachial plexus injury were associated with fractures classified as birth-related as well as in the yearly period (< 6 months) of humerus shaft fracture. The major weakness of the study was that we did not have information from patient records regarding treatment and cause of injury. This also limited our analysis of the clinical circumstances of shaft fractures registered as both birth-related (P coded) and other during infancy (S coded), and of shaft fractures in the early neonatal period not diagnosed as birth-related. This also underlines the importance of addressing shaft fractures whether accidental or non-accidental by a population case-control study with access to hospital records which is in planning stage. Furthermore, the extent of testing for biochemical markers or genetic disease could not be determined, meaning that there might have been cases with an abuse diagnosis disorder in which proper differential diagnostics had not been undertaken.

## Conclusion

This study is the largest population-based report and provides data on epidemiology, birth, parental characteristics, and reported accidents in relation to femur and humerus shaft fractures during infancy and may not only serve as a basis for further studies but this may also be of clinical importance especially for health professionals in training. The present study identified reported accidents as the main contributor to femur and humerus shaft fractures during infancy, a considerable proportion associated to abuse diagnosis, and that bone fragility factors and birth trauma might be of importance not only for fractures diagnosed as birth-related. The incidence of birth-related fractures differs substantially from previously reported hospital case studies. We recommend pediatricians and pediatric orthopedic surgeons to use double coding, i.e., adding S codes to P codes, for improved precision of the registration of birth-related fractures.

## Supplementary Information

**Additional file 1: Table S1.** Definitions of fractures, accidents, and others. Swedish version of the International Statistical Classification of Diseases (ICD-10-SE).

## Data Availability

The datasets used and/or analysed during the current study are available from the corresponding author on reasonable request.

## Abbreviations

NPR: National Patient Register
SMBR: The Swedish Medical Birth Register
RCWM: The Register of Children and Young Persons Subjected to Child Welfare Measures
ICD-10-SE: Swedish version of International Classification of Diseases
SGA: Small-for-gestational age
LGA: Large-for-gestational age
BMI: Body mass index
OI: Osteogenesis imperfecta
ALP: Alkaline phosphatase
PTH: Parathyroid hormone
