# Supplementary material for: The epidemiology of infant shaft fractures of femur or humerus by incidence, birth, accidents, and other causes
Source: BMC Musculoskelet Disord. 2020 Dec 11;21:840. doi: 10.1186/s12891-020-03856-4 (PMC7731463; doi:10.1186/s12891-020-03856-4)
Supplement: Supplementary file 1 — Additional file 1: Table S1. Definitions of fractures, accidents, and others. Swedish version of the International Statistical Classification of Diseases (ICD-10-SE). [file 12891_2020_3856_MOESM1_ESM.docx]

**TABLE S1.** Definitions of fractures, accidents, and others. Swedish version of the International Statistical Classification of Diseases (ICD-10-SE).

| **Category** | **Diagnosis** | **ICD-10 codes** |
| --- | --- | --- |
| Fracture |  |  |
|  | Fracture of shaft of femur | S72.3 |
|  | Fracture of neck of femur | S72.0 |
|  | Pertrochanteric fracture | S72.1 |
|  | Subtrochanteric fracture | S72.2 |
|  | Fracture of lower end of femur | S72.4 |
|  | Fracture of other parts of femur | S72.8 |
|  | Fracture of femur, part unspecified | S72.9 |
|  | Fracture of shaft of tibia | S82.2 |
|  | Fracture of fibula alone | S82.4 |
|  | Fracture of medial malleolus | S82.5 |
|  | Fracture lateral malleolus | S82.6 |
|  | Fracture of shaft of humerus | S42.3 |
|  | Fracture of humerus upper end | S42.2 |
|  | Fracture of humerus lower end | S42.4 |
|  | Fracture of clavicle | S42.0 |
|  | Fracture of upper end of ulna | S52.0 |
|  | Fracture of upper end of radius | S52.1 |
|  | Fracture of shaft of ulna | S52.2 |
|  | Fracture of shaft of radius | S52.3 |
|  | Fracture of shaft of both radius and ulna | S52.4 |
|  | Fracture of lower end radius | S52.5 |
|  | Fracture of lower end of both radius and ulna | S52.6 |
|  | Multiple fractures of forearm | S52.7 |
|  | Fracture of other parts of forearm | S52.8 |
|  | Rib fracture | S22.3, S22.4 |
| Birth injury to skeleton |  |  |
|  | Birth injury to other long bones | P13.3 |
|  | Birth injury to femur | P13.2 |
|  | Fracture of clavicle due to birth injury | P13.4 |
| Accidental injury |  |  |
|  | Transport accident | V01-99 |
|  | Fall accidents | W00-19 |
|  | Same level | W01 |
|  | While being carried | W04 |
|  | Bed | W06 |
|  | Chair or other furniture | W07, W08 |
|  | Playground equipment | W09 |
|  | Stairs and steps | W10 |
|  | Ladder | W11 |
|  | Pinch accidents | W23, W52 |
| Others |  |  |
|  | Brachial plexus birth injuries | P14.0, P14.1, P14.3 |
|  | Dystocic labor | O62-O67 |
|  | Osteogenesis imperfecta | Q78.0 |
|  | Rickets or Vitamin D deficiency | E55.0, E55.9 |
|  | Infant abuse diagnosis (observation for suspected abuse, battered baby syndrome, maltreatment syndrome) | Z03.8K, Y07, T74.1, Y06 |
|  |  |  |
